# Insecticide-Treated Nets Utilization and Associated Factors among under-5 Years Old Children in Mirab-Abaya District, Gamo-Gofa Zone, Ethiopia

**DOI:** 10.3389/fpubh.2018.00007

**Published:** 2018-02-01

**Authors:** Amha Admasie, Amanuel Zemba, Wondimagegn Paulos

**Affiliations:** ^1^School of Public Health, College of Health Sciences and Medicine, Wolaita-Sodo University, Wolaita-Sodo, Ethiopia

**Keywords:** malaria, insecticide-treated nets, utilization, under-5 years old children, Mirab Abaya

## Abstract

**Background:**

Malaria can be prevented using cost-effective interventions. It can be prevented at large *via* the use of insecticide-treated mosquito nets (ITNs). The use of ITNs decreases malaria mortality rates by 55% in under-5 years old children in Africa, Ethiopia, realizing the effectiveness, scaling up distribution and utilization of ITNs to cover 100% of children less than 5 years of age. However, little is known about ITNs utilization and factors associated with the utilization in under-5 years old children in the study area yet. The purpose of this study was to assess the level and associated factors of ITNs utilization in under-5 years old children among households with under-5 years old children of Mirab Abaya District, Gamo Gofa Zone, Ethiopia.

**Methods:**

A community-based cross-sectional study was conducted during August–September, 2016. Six study Kebeles were identified by simple random sampling technique and 398 households with at least one under-5 years old children were selected by random sampling technique using computer generated random numbers from health post family folders. Structured, interviewer questionnaire was administered to mothers or care givers of the children. Data were entered to Epi Info Version 3.5 and analyzed in SPSS version 21 statistical software. Bivariate and multivariate logistic regression analysis was done. *P*-value <0.05 and odds ratio with 95% confidence interval were used for the determination of associations between dependent and predictor variables. Results were presented in narrations, tables, and graph.

**Result:**

Among 398 under-5 years old children assessed, the majority, 362 (91.0%) of them had access to ITN, but only 137 (37.2%) of the child had ITNs utilization during the previous night prior to the survey. Households with age of mothers or caretakers 31–44 years, AOR = 0.03, 95% CI (0.01–0.07) and ≥45 years of age; AOR = 0.05, 95% CI (0.01–0.58); households with family size ≤5 members, AOR = 11.23, 95% CI (4.31–29.24); and households with sleeping space ≥2, AOR = 13.59, 95% CI (4.40–41.93) were found to be significantly associated with under-5 years old children ITNs utilization.

**Conclusion:**

Even though, a significant proportions of under-5 years old children had access to ITN, only one-third of the participant child utilized it properly.

## Introduction

According to the reports of World Health Organization, nearly 3.3 billion people in 97 countries are potentially at risk of malaria (1). Globally in the year 2015, an estimated 214 million cases of malaria and 438,000 malaria deaths were enumerated (2). The problem of malaria in the African Region is devastating and causing 78% of all deaths in under-5 years old children is due to malaria cases (1, 2). Fifty seven million (68%) of Ethiopians live in malaria endemic area (3).

The use of insecticide-treated nets (ITNs), indoor residual spraying, and case management are cost-effective interventions of malaria (2). The distribution and use of ITNs is one of the main interventions for malaria infection prevention in developing countries (4). Hence, policies of promoting universal access to ITNs are developed in most malaria endemic states (2). But, the proportion of the population slept under ITNs in 2015 in Sub-Saharan Africa was estimated to be 55%; and 68% of them were under-5 years old children (2). Recently, Ethiopia is working for the effectiveness of ITNs for prevention of malaria transmission, scaling up distribution and utilization of ITNs to cover 100% of under-5 years old children (5). Ethiopia’s national policy of malaria prevention aims to provide one ITNs for every sleeping space (approximately one net per 1.8 persons in malaria-endemic areas of <2,000 m altitude) (4). Recent researches indicated that the proper utilization of ITNs among high risk groups were found to be very low, and it also revealed that increase in ITNs access does not necessarily indicate equal increase in ITN utilization (6). The Ethiopia National Malaria Strategic Plan recognizes that the use of ITNs is a key strategy to roll back malaria and free distribution of ITNs in every 3 years to all peoples living in endemic, high, and moderate malaria risk areas of Ethiopia (3). Two ITNs per household was used as an operational guideline until 2011, but that policy was then changed to one ITNs per 1.8 persons through mass and free of charge distribution campaigns to achieve universal coverage (3).

Under-5 years old children and pregnant women are vulnerable to malaria and are targeted as high priority groups for ITNs distribution in the country (3, 7). Therefore, households in targeted areas of vulnerable groups have higher chance of getting free of charge ITNs. Despite the rapid and complete coverage of ITNs distribution, reports indicated that still there is very poor ITNs utilization (7).

The Ethiopia national malaria strategic plan 2011–2015 had a target to achieve 100% of households in malaria endemic areas to own at least one ITN per sleeping space at least in 80% of malaria risk population (8). Later, the Ethiopia National Malaria Strategic Plan 2014–2020 had targeted to provide ITNs to all population groups living in endemic areas to reach and maintain 100% ownership and utilization of ITNs (5).

The most recent, large, and nationally representative Ethiopia malaria indicator survey result showed that 55.2% of households have at least one mosquito net and the under-5 years old children utilizations of ITNs were 38.2%. According to this report, ITNs ownership and utilization of under-5 years old children in Southern, Nation, Nationalities and Peoples Region (SNNPR) of Ethiopia was 57 and 42%, respectively (4). According to studies conducted in different areas of Ethiopia, household ownership of at least one ITNs and ITNs utilization among under-5 years old children were 25.3 and 63.9%, respectively, at Adami Tulu district of Oromia region (9), 62.4 and 21.5%, respectively, at Gursum district of Oromia region (10), 95.8 and 59.1%, respectively, in Dejen Woreda, Amhara region (11), 98.4 and 69%, respectively, in Arbaminch Zuria woreda of SNNPR (6), 44.96 and 32% respectively, Shebedino district, SNNPR (12), 71.64% of utilization, Raya Alamata District of Amhara region (13), ownership was 56.6% in Gilgel-Gibe, Ethiopia (14), in SNNPR, 67.5% of households had used a bed net during the previous night. But, only 9.4% were used by children alone (15).

A 2-years’ prospective cohort study done at Arba Minch Zuria Woreda, Gamo Gofa Zone, Ethiopia showed that the ITNs use fraction reached to 69%, but the utilization in under-5 years old children was very low (6).

Knowing the factors affecting ITNs utilization is essential for consistent and efficient use of it. Hence, knowing that ITNs kills malaria mosquitoes (9), households whose family size was less than or equal to four (10), household with more than one sleeping room (16), female heads (17), male heads (18, 19), gender (20) were associated with ITNs utilization among under-5 years old children. Many studies in Africa had showed similar findings (17, 20, 21). However, a study done in Burkina Faso had shown no significant difference between ITNs utilization and family size (22) and sharing of sleeping space increases ITNs utilization in Nigeria (20).

In Ghana, reasons for not utilizing ITNs were absence/low number of mosquitoes, malaria was not their health problem concern, felt discomfort sleeping under ITNs, less quality of ITNs, and improper ITNs placement (23). In Nigeria, house-types, mothers’ occupation, mother’s educational level, and ITNs ownership were significant factors of ITNs utilization (24). In Ethiopian context, age of mother, being female, increasing proportion of good quality bed nets and increasing net density were significant determinants of ITNs utilization (25).

Most studies in Ethiopia depicted that the rate of ITNs utilization is very low among under-5 years old children, which is against to the national target. Even though various predictors were studied, but certain sociodemographic and intra household characteristics as predictor of ITNs utilization to under-5 years old children have not been addressed well. Scientific evidence is needed to uncover and support possible associations between these selected sociodemographic and intra-household characteristics and ITNs utilization among under-5 years old children to prevent malaria. Therefore, the objective of this study was to assess ITNs utilization in under-5 years old children and associated factors among households with under-5 years old children in Mirab Abaya District, Gamo Gofa Zone, Southern Ethiopia.

## Materials and Methods

### Study Setting

This study was conducted in Mirab Abaya District, Gamo Gofa Zone, Southern Ethiopia from August to September, 2016. The town of the district, Birbir town, is located 450 km from Addis Ababa, in the Southwest direction, along the way to Arba Minch town. Geographically, the district is located at an altitude between 1,100 and 2,900 m above sea level. Administratively, it is divided in to 24 Kebeles (one urban and 23 rural). The total population of the district in 2016 is 93,459, of which, about 14,589 (15.4%) were estimated to be under-5 years old children. The district has three major agro-ecologies. Population distribution in low land agro-ecologic zones is 67,229 (72%) and out of the 24 Kebeles, 16 are in the (lowland) agro-ecology, below 2,000-m altitude. In the low land, average annual rainfall ranges from 800 to 1,600 mm.

The district has 4 health centers with 26 satellite health posts. Malaria is seasonal particularly from September to December and occurs in epidemic type, and *plasmodium falciparum* (about 70%) is a dominant malaria species. The main source of income of the residents is agriculture, which is supported by an irrigation system using engine pump technology from Lake Abaya.

### Study Design and Population

A community-based cross-sectional study was used supplemented with observation methods. The study population was all under-5 years old children in Mirab Abaya District, Gamo Gofa Zone, Southern Ethiopia who were randomly selected households with at least one under-5 years old child in the household. But under-5 years old children who do not stay in the household the night before the interviewer’s visit were excluded. Index child (the youngest child in the household) was taken as a study participant. Household data were collected from mothers or caretakers of the index child. ITNs utilization of under-5 years old children was assessed based on mothers’ or caretakers’ self-report of ITNs utilization of the previous night of the survey.

### Sample Size

The required sample size for primary objective was calculated using Open Epi, Version 3.03, software package based on the assumptions: single population proportion for cross-sectional survey assuming proportion (P) of 42% children had slept under ITNs in the previous night of the survey in SNNPR (4). Assuming 5% margin of error at 95% confidence level. The final sample size with 10% non-response rate was 413.

### Sampling Procedures

The district has 24 Kebeles, and 6 Kebeles were selected using simple random sampling method and then, and the number of households for each selected 6 Kebeles were determined by population proportion to size principles (16, 26). Since the study units are under-5 years old children, the number of households, which were included in the study in each selected Kebeles were proportional to the total number of households (26). Participant households were selected by systematic random sampling method using computer generated random number from family folder list of households. When the randomly selected household do not fulfill the inclusion criteria, the immediate neighborhood household that fulfill the inclusion criteria was interviewed.

### Data Collection Methods and Quality Checks

Data were collected using face-to-face administered structured questionnaire, which was adapted from Ethiopia Malaria Indicator Survey 2011 and other different published literatures (4, 9, 21, 27), and the questionnaire includes questions about the respondent’s sociodemographic characteristics, intra-household factors, households ITNs ownership, and utilization of the under-5 years old children in the household during the night preceding the survey.

The questionnaire was prepared in English and then translated to Amharic in consultation with language professionals and translated back to English to check its consistency.

Four nurses from public health centers with prior experience in data collection and who can speak one of the local languages Wolaitatho, Gamotho, or Gidicho fluently were recruited for data collection. Two public health officers were assigned as supervisors. Two days of training was given to data collectors and supervisors.

The questionnaire was pretested in 20 households (5% of the total sample) at Wanke Kebele, which is not included in the study. Questionnaires were checked for completeness at the time of data collection. Feedbacks on previous day activities were given for both data collectors and supervisors on daily basis.

### Variables

The dependent variables were insecticide-treated nets (ITNs) utilization of under-5 years old children and the predictor variables were sociodemographic characteristics and intra-household factors: number of ITNs in the household, family size, number of bed rooms, and number of beds/sleeping space.

### Operational Definitions

#### Household Ownership of ITNs

Households are considered as ITNs owners if they have at least one ITN at the time of the interview (9).

#### ITNs Utilization

A child (index child) who was reported to have slept under ITNs during the night prior to the survey date (3–5, 28).

#### Caretakers

A household member who takes care of under-5 years old children and of age above 18 years old.

### Data Management and Analysis

Data were checked for completeness, consistency, accuracy, and comprehensiveness. Data were coded and entered in to Epi-info version 3.5 computer software package. Cleaned data were exported to SPSS (statistical package for social sciences) software version 21 for statistical analysis. Both bivariate and multivariable analysis were run. Odds ratio with 95% CI, and *P*-value less than 0.05 were used for both analyses. The Hosmer–Lemeshow test of Good-fit model was tested and multi-collinearity test before multivariate analysis were checked for the presence of multi-collinearity between independent variables.

### Ethical Consideration

Ethical clearance was obtained from Wolaita Sodo University, College of Health Science and Medicine, School of Public Health, Institutional Review Board. Study participants were told about the objectives and aims of the study in detail, and all participants were asked for their consent to be involved in the study and they were informed that their participation is voluntarily. Confidentiality was assured to all participants. Data collectors put their signature for they had informed verbal consent for the interview and the observation done. Children who were febrile during data collection time were referred to the nearby health post.

## Results

### Sociodemographic Characteristics of the Households

A total of 398 households with under-5 years old children were participated in the study with 96.35% response rate. Majority, 387 (97.2%) of the household heads were females. The mean age of the mothers or caretakers was 35 years with SD of ±7.82 years. Regarding educational status, 142 (35.7%) of the mothers/caretakers were illiterate. Above half (51.3%) of the mothers/caretakers were from Gamo ethnic group. The main occupation of the household head was farming, 360 (90.5%). Fifty-nine percent of the children were living in a family of more than five families in the house (Table 1).

**Table 1:** Sociodemographic characteristics of households, Mirab Abaya District, Gamo Gofa Zone, Ethiopia, August–September, 2016.

Characteristics	Response (*n* = 398)	Frequency	%
Sex of HH head	Male	387	97.2
	Female	11	2.8

Age of mothers/caretakers	18–30 years	149	37.4
	31–44 years	228	57.3
	≥45 years	21	5.3

Educational states of mothers/caretakers	Illiterate	142	35.7
	Literate	256	64.3

Religion of caretakers	Protestant	362	91.0
	Orthodox	36	9.0

Ethnic group caretakers	Gamo	204	51.2
	Wolaita	92	23.1
	Gidicho	95	23.9
	Others	7	1.8

Marital status mothers/caretakers	Married	385	96.7
	Others	13	3.3

Occupation of household head	Farmers	360	90.5
	Non farmer	38	9.5

Family size in the household	≤5	163	41.0
	>5	235	59.0

### Intra-Household Characteristics of the Households

Majority, 360 (90.5%) of households had only one separate bed room. The number of beds/sleeping spaces, 238 (59.8%) of households had greater or equal to two sleeping spaces. 368 (92.5%) of the households had ITN in their households, but the number of beds/sleeping spaces observed with ITNs mounted over the bed in the households were only 158 (42.9%). Even though, 362 (91.0%) of the under-5 years old children had access to ITN, only 137 (37.2%) under-5 years old children were utilized ITNs in the previous night of the survey (Table 2).

**Table 2:** Intra-household characteristics of the households with under-5 years old children, Mirab Abaya district, Gamo Gofa Zone, Ethiopia, August–September, 2016.

Characteristics	Response	Frequency	Percent
Number of bed rooms (*n* = 398)	1	360	90.5
	≥2	38	9.5

Number of beds/sleeping space (*n* = 398)	1	160	40.2
	≥2	238	59.8

Availability of insecticide-treated mosquito nets (ITNs) (*n* = 398)	Yes	368	92.5
	No	30	7.5

Number of ITNs reported (*n* = 368)	1 ITN	111	30.2
	2 ITN	182	49.5
	≥3 ITN	75	20.3

Number of ITNs observed (*n* = 398)	Yes	362	91.0
	No	36	9.0

Number of ITNs observed (*n* = 362)	1 ITN	205	56.6
	2 ITN	109	30.1
	≥3 ITN	48	13.3

ITNs mounted over the bed (*n* = 368)	Yes	158	42.9
	No	210	57.1

Under-5 years old children utilized ITN (*n* = 368)	Yes	137	37.2
	No	231	62.8

### ITNs Accessibility

**Figure 1:**
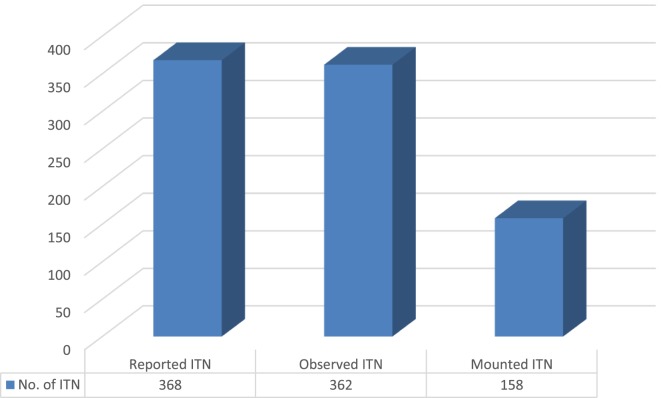
The number of insecticide-treated mosquito nets (ITNs) reported, observed, and mounted in the households of Mirab Abaya District, Gamo Gofa Zone, Southern Ethiopia, August–September, 2016.

Among 398 households with at least one under-5 years old children, 362 (91.0%) households with under-5 years old children had at least one ITNs in the household, whereas 36 (9.0%) were no ITNs in the household. The number of ITNs of self-reported, observed by data collectors and mounted over the bed had great variation in number (Figure 1).

### Factors Associated with ITNs Utilization in Under-5 Years Old Children

Factors associated with not utilizing ITNs in under-5 years old children were hotness during sleeping under ITNs, absence of mosquito or malaria at the survey time, rectangular shape ITNs inappropriate to mount, and insufficient ITNs. Most households were using ITNs for unintended purposes like raping over mattress to protect from bugs, for grain and fruit carrying, to spread grains on the sunshine, using as screen/curtain, for fishing, and other similar purposes were observed during the survey (Table 3).

**Table 3:** Factors associated with insecticide-treated mosquito net (ITN) utilization among under-5 years old children of Mirab Abaya District, Gamo Gofa Zone, August to September, 2016.

Variable	Category	ITN utilization		COR (95% CI)	AOR (95% CI)
		Yes	No		
Age of mothers	18–30 years	125	24	1.00	1.00
	31–44 years	11	217	0.01 (0.00–0.02)	0.03 (0.01–0.07)
	≥45	1	20	0.01 (0.00–0.07)	0.05 (0.00–0.58)

Educational status	Illiterate	10	132	1.00	1.00
	Literate	127	129	13.0 (6.53–25.86)	2.02 (0.63–6.46)

Occupation status	Farmer	131	229	3.05 (1.24–7.49)	2.96 (0.56–15.56)
	Non-farmer	6	32	1.00	1.00

Family size	≤5 families	122	41	43.64 (23.21–82.06)	11.23 (4.31–29.24)
	>5 families	15	220	1.00	1.00

No. under-5 years old	1	70	105	1.55 (1.02–2.35)	0.62 (0.23–1.69)
	≥2	67	156	1.00	1.00

No. Bed rooms	1	102	258	1.00	1.00
	≥2	35	3	29.51 (8.88–98.09)	0.46 (0.07–3.31)

No. sleeping space	1	7	153	1.00	1.00
	≥2	130	108	26.31 (11.83–58.52)	13.59 (4.40–41.93)

## Discussion

Insecticide-treated nets is one of the most important mechanisms that provide a feasible and effective means of combating malaria in highly endemic areas (9) like Mirab Abaya district. Though the Ethiopian Federal Ministry of Health works substantially to increase ownership of ITNs in each malaria endemic areas, but, many barriers have been indicated in terms of proper utilization of ITNs.

This study identified that only 37.2% of under 5 years of age children utilized ITNs the previous night prior to the survey. The finding was in line with slight difference with the study from Southern Ethiopia, which was 27.2% (15). However, it was boldly lower than a study conducted in malaria endemic areas of Raya Alamata District, Ethiopia 71% and in Burkina Faso, 70% (13, 22). The possible explanation for such discrepancies could be geographical variations, the time gap between distributions of ITNs, and survey period and also other age groups might be utilized as evidenced in these studies (6).

The level of utilization was not acceptable as it falls far below the 80% target of Ethiopia Malaria Strategic Plan (2011–2015) for these risk groups, even though we are beyond the plan period (3, 8). Also the prevalence of under-5 years old children, ITNs utilization in this study was far below 2011 MIS result of SNNPR, Ethiopia, for an acceptable level of protection (4).

Households with mothers/caretakers age group of 31–44 and 45 years and above were less likely to have their under-5 years old children utilized ITNs than those in the age range of 18–30. The result was in agreement with the findings from Kenya and Nigeria (17, 20). This could be explained as young age groups are more educated than old aged as evidenced on a study from Nigeria (20), and also it could be explained by the theory of adaptation to innovations of social psychology, which maintains that younger people are more likely to be innovators and early adaptors of new technology may be at work here (20).

Households with family size of ≤5 members were more likely to have their under-5 years old children utilized ITNs than those with >5 members. This is consistent with findings from Ethiopia and Kenya (10, 17). In contrary from Ethiopian and Kenyan study evidences, a study conducted at Burkina Faso showed that family size showed no significant difference in under five ITNs utilization (22). The possible explanations for this might be in proportionate family size with the number of bed rooms or separate sleeping space. Using ITNs in single room houses could be a challenge due to lack of convenient space, which does not permit mosquito net hanging as evidenced by the study from Uganda and Ethiopia. In addition, the sleeping room could serve for other household activities. Also, this could be attributed to the availability of ITNs, since it is easier for few members of a household to share the ITNs as compared with many household members with few ITNs (14, 21).

Households with number of beds or separate sleeping space ≥2 were more likely to utilize ITNs for their under-5 years old children than those with number of beds or separate sleeping space of one room. The study conducted in Kersa, Eastern Ethiopia, was in agreement with this study (16). But, a study from Nigeria was contrary to our result, bed/sleeping space sharing of children with other family members increase ITNs utilization (20). Possible assumption for these could be sharing of one sleeping place for more than one person in the house could not be convenient to use ITNs for all members of the household as seen in the study from Kersa, Eastern Ethiopia (16).

### Strengths and Limitations of the Study

Observation of self-reported ITNs and ITNs mounted over the sleeping area during the interview were the strengths of this study. However, the study was conducted based on apparently small sample size use of ITNs. Most of the responses were self-reported, which was subjected to social desirability bias. Due to the nature of cross-sectional design, it could lack its ability to establish a cause and effect relationship between determinants and outcome. Despite these limitations, this study contributed a different perspective to current knowledge and potential implications about ITNs utilization in under-5 years old children in the study district and in the country.

## Conclusion

This study showed that only about one-third of under-5 years old children utilized ITNs during the night prior to the survey. The mother’s or care taker’s age being greater than 30 years, family size of greater than 5 years, and households with number of beds/sleeping space only one sleeping space were significant factors of under-5 years old children for lower ITNs utilization.

### Recommendations

All primary health care units (PHCUs) in the district, health extension workers, women’s development army, and one to five networks leaders should conduct community sensitization campaign on ITNs mounting and utilization especially on under-5 years old children. Continuous and strengthened awareness creation sessions by PHCUs on ITNs utilization of under-5 years old children should be maintained in immunization sessions, under five clinics, and in any population gatherings concurrently. Integrated family planning education should be considered in the family for better ITN utilization. As a long-term plan, the local government should devise standard housing program so that each family member would sleep in a separate sleeping space.

## Author Contributions

AZ designed the study protocol and is involved in data collection and analysis. All authors involved on protocol development. AA and WP are involved in data analysis, supervised and monitored the protocol. AA prepared the manuscript, and WP edited the manuscript. All authors are involved on protocol development, read and approved the final manuscript.

## Conflict of Interest Statement

The authors declare that the research was conducted in the absence of any commercial or financial relationships that could be construed as a potential conflict of interest.
